# Plasma miRNA-Metabolite Dysregulation in People with HIV with Cirrhosis Despite Successful HCV Cure

**DOI:** 10.3390/ph19010170

**Published:** 2026-01-19

**Authors:** Ana Virseda-Berdices, Raquel Behar-Lagares, Juan Berenguer, Juan González-García, Belen Requena, Oscar Brochado-Kith, Cristina Díez, Victor Hontañon, Sergio Grande-García, Carolina González-Riano, Coral Barbas, Salvador Resino, Amanda Fernández-Rodríguez, María Ángeles Jiménez-Sousa, the Marathon Study Group

**Affiliations:** 1 Unidad de Infección Viral e Inmunidad, Centro Nacional de Microbiología (CNM), Instituto de Salud Carlos III (ISCIII), 28222 Majadahonda, Spain; anavirseda@externos.isciii.es (A.V.-B.); raquelbehar82@gmail.com (R.B.-L.); amandafr@isciii.es (A.F.-R.); 2 Centro de Investigación Biomédica en Red en Enfermedades Infecciosas (CIBERINFEC), Instituto de Salud Carlos III (ISCIII), 28029 Madrid, Spain; jbb4@me.com (J.B.); juangonzalezgar@gmail.com (J.G.-G.); brochado1993@gmail.com (O.B.-K.); crispu82@gmail.com (C.D.); victor.hontanon@gmail.com (V.H.); sergio.grande@isciii.es (S.G.-G.); 3 Facultad de Medicina, Universidad Complutense de Madrid, 28040 Madrid, Spain; 4 Unidad de Enfermedades Infecciosas/VIH, Hospital General Universitario “Gregorio Marañón”, 28007 Madrid, Spain; 5 Instituto de Investigación Sanitaria Gregorio Marañón (IiSGM), 28007 Madrid, Spain; 6 Servicio de Medicina Interna-Unidad de VIH, Hospital Universitario La Paz, 28046 Madrid, Spain; 7 Instituto de Investigación Sanitaria La Paz (IdiPAZ), 28046 Madrid, Spain; 8 Centro de Metabolómica y Bioanálisis (CEMBIO), Facultad de Farmacia, Universidad San Pablo-CEU, 28668 Boadilla del Monte, Spain; belen.fernandezrequena@ceu.es (B.R.); carolina.gonzalez@isciii.es (C.G.-R.); cbarbas@ceu.es (C.B.); 9 Facultad de Ciencias de la Salud, Universidad Rey Juan Carlos, 28922 Alcorcón, Spain; 10 Unidad de Patogénesis e Inmunidad Viral, Centro Nacional de Microbiología (CNM), Instituto de Salud Carlos III (ISCIII), 28220 Majadahonda, Spain

**Keywords:** HIV, HCV clearance, miRNA profile, cirrhosis, metabolomics

## Abstract

**Background**: Persistent liver pathology despite a sustained virologic response (SVR) to hepatitis C virus (HCV) therapy is a major clinical concern. This is particularly relevant for people with HIV (PWH) with HCV coinfection, a population prone to accelerated liver disease progression. This study aimed to characterize the plasma miRNA profile in PWH with cirrhosis one year after successful completion of HCV therapy, and to explore their relationship with metabolite alterations. **Methods**: This cross-sectional study enrolled 47 PWH who achieved HCV clearance with antiviral therapy. Using plasma samples collected approximately one year after completion of HCV therapy, participants were stratified into two groups based on liver stiffness measurement (LSM): compensated cirrhosis (*n* = 32, LSM ≥ 12.5 kPa) and non-cirrhosis (*n* = 15, LSM < 12.5 kPa). Plasma miRNAs and metabolites were determined using small RNA sequencing and untargeted capillary electrophoresis-mass spectrometry (CE-MS), respectively. Significantly differentially expressed (SDE) miRNAs were identified using generalized linear models (GLM) with a negative binomial distribution, and their correlation with metabolite levels was quantified using Spearman’s correlation. **Results:** In the cirrhosis group (*n* = 32), we identified a distinct signature of 15 SDE miRNAs (9 upregulated, 6 downregulated) compared to the non-cirrhotic group (*n* = 15), showing hsa-miR-10401-3p, hsa-miR-548ak, hsa-miR-141-3p, and hsa-miR-3940-3p the largest expression changes. miRNA-gene interaction and pathway enrichment analysis suggested that these 15 SDE miRNAs potentially regulate multiple genes involved in immune response and amino acid metabolism. In addition, correlation analyses with our metabolomic data revealed significant associations between specific SDE miRNAs and amino acids and their derivatives. Specifically, the expression of upregulated miRNAs (e.g., hsa-miR-10401-3p and hsa-miR-16-5p) was positively correlated with plasma levels of L-methionine and its derivatives, while downregulated miRNAs (e.g., hsa-miR-625-5p) were inversely correlated with L-tryptophan. **Conclusions**: In cirrhotic PWH with history of HCV coinfection, a distinct plasma miRNA signature linked to dysregulated amino acid metabolism is found one year after completion of HCV therapy. This underscores that the HCV cure does not equate to complete hepatic recovery, highlighting the critical need for long-term monitoring in this high-risk population.

## 1. Introduction

The introduction of direct-acting antivirals (DAAs) has revolutionized treatment, enabling remarkable sustained virologic response (SVR) rates above 95% [1]. However, SVR does not always guarantee complete resolution of underlying liver injury. Patients, particularly those with pre-existing advanced fibrosis or cirrhosis, remain at risk of complications such as liver failure and hepatocellular carcinoma (HCC) even after achieving SVR [2]. These persistent hepatic alterations suggest that molecular drivers of damage may remain active despite viral clearance. In addition, in people with HIV (PWH), liver disease is a leading cause of non-AIDS-related mortality [3], with risk further amplified in those with a history of HCV coinfection due to complex virus–host interactions. Elucidating the underlying molecular mechanisms is crucial for improving long-term patient care and circulating microRNAs (miRNAs) offer a promising investigative tool.

MiRNAs are small non-coding RNA molecules that primarily regulate gene expression by binding to messenger RNA (mRNA) targets, leading to either the inhibition of protein translation or the promotion of mRNA degradation [4]. Their stability in body fluids, such as like blood [5], makes them excellent non-invasive biomarkers for the diagnosis, monitoring, and prognosis of liver diseases [6]. Beyond their role as biomarkers, miRNAs are critical regulators of fundamental biological processes, including metabolic pathways that are profoundly disrupted in chronic liver disease [7,8]. In the context of HCV-infected patients, particularly those with persistent liver damage post-SVR, miRNAs may reflect ongoing cellular stress, metabolic alterations, and signalling pathways that perpetuate liver injury. This makes them highly relevant for identifying residual pathological activity that is not captured by standard clinical assessments.

Both HIV and HCV can hijack host cellular miRNA machinery, leading to dysregulated miRNA profiles in mono- and coinfection [9,10], with some miRNAs even playing a role in HCV and HIV replication [11]. For instance, miR-122, miR-181, miR-155, miR-29, and miR-223 are dysregulated in HIV/HCV coinfected patients [4]. In addition, Anadol et al. showed higher levels of miRNA-122, miRNA-34a, and miRNA-22 in serum samples from PWH with liver injury compared to PWH with other conditions [12]. Importantly, specific plasma miRNA profiles can predict liver fibrosis progression in chronically HIV/HCV-coinfected patients who did not yet exhibit liver fibrosis at the time of sampling [2].

Despite this growing body of evidence, the miRNA expression profile in PWH with advanced liver disease after HCV eradication remains largely uncharacterized. Therefore, this study aimed to characterize plasma miRNA associated with compensated cirrhosis in PWH one year after successful HCV eradication and to explore their relationship with metabolite alterations.

## 2. Results

### 2.1. Patient Characteristics

Of the initial cohort of PWH who achieved SVR after HCV therapy, 47 patients were included in this cross-sectional study one year after HCV therapy completion; the remaining patients were not included due to unavailability of LSM data at the study time point (*n* = 14), or lack of available miRNA and metabolomic data (*n* = 2). Clinical and epidemiological characteristics of the 47 PWH enrolled in the study are detailed in Table 1.

The cohort was stratified into a cirrhosis group (*n* = 32, 68.1%) and a non-cirrhosis group (*n* = 15, 31.9%). The study population was exclusively of Caucasian origin. The two groups were well-matched, showing no significant differences in age, gender distribution, body mass index, hepatic steatosis index, tobacco use, or alcohol consumption. As expected by the study design, the cirrhosis group had significantly higher LSM values (median 18.6 kPa vs. 7.2 kPa, *p* < 0.001). We also observed a significant difference in the type of HCV therapy received between the groups (*p* < 0.001).

### 2.2. MiRNome Characterization in PWH with Cirrhosis

On average, 15 million reads were obtained per sample, exceeding the minimum depth required for robust miRNA expression analysis [13]. Out of a total of 2656 detected miRNAs (representing 76.2% of the miRBase annotated miRNome), 380 miRNAs passed expression filtering criteria and were retained for differential expression analysis. PCA confirmed the absence of significant batch effects across the sequencing runs (Supplementary Data S4). Raw sequencing data are available in the BioStudies repository under accession code S-BSST2284 [14].

**Figure 1:**
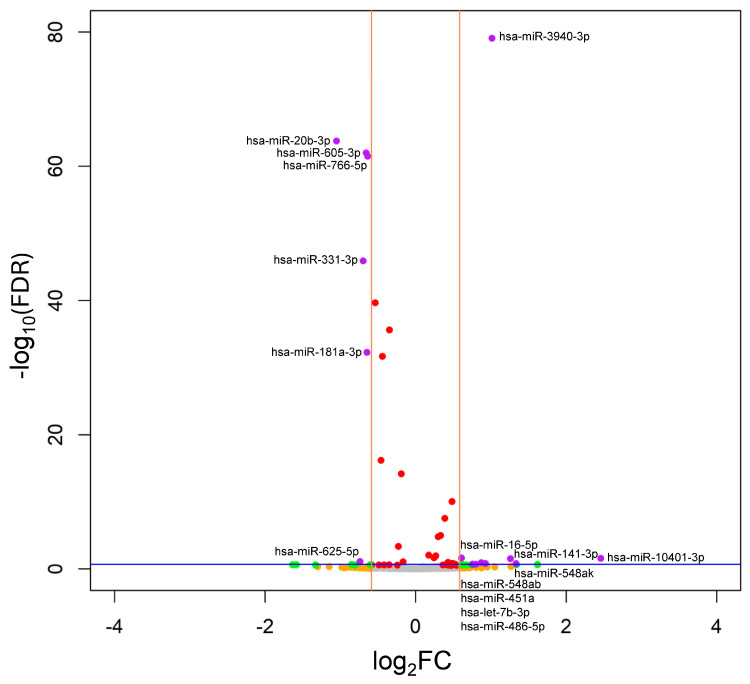
Volcano plot of differential miRNA expression between PWH with and without cirrhosis one year after successful completion of HCV therapy. The vertical orange lines indicate the log_2_FC threshold of ±0.585, and the horizontal blue line indicates the FDR significance threshold of 0.2. Each dot represents a single miRNA. Purple dots show miRNAs with a |log_2_FC| ≥ 0.585 and FDR-corrected *p*-value ≤ 0.2; green dots show miRNAs with a |log_2_FC| ≥ 0.585 and FDR-corrected *p*-value > 0.2, red dots show miRNAs with FDR-corrected *p*-value ≤ 0.2 but with |log_2_FC| < 0.585 and orange dots represent miRNAs with a |log_2_FC| ≥ 0.585 that do not present statistical significance; grey dots show miRNAs without statistical significance and a |log_2_FC| < 0.585. **Abbreviations**: FDR, false discovery rate; log_2_FC, log_2_ fold change.

Differential expression analysis between the cirrhosis and non-cirrhosis groups identified a distinct signature of 15 SDE-miRNAs (Figure 1). Of these, nine miRNAs were significantly upregulated (hsa-let-7b-3p, hsa-miR-10401-3p, hsa-miR-141-3p, hsa-miR-16-5p, hsa-miR-3940-3p, hsa-miR-451a, hsa-miR-486-5p, hsa-miR-548ab, and hsa-miR-548ak), while six were downregulated in patients with cirrhosis (hsa-miR-181a-3p, hsa-miR-20b-3p, hsa-miR-331-3p, hsa-miR-605-3p, hsa-miR-625-5p, and hsa-miR-766-5p). Notably, four upregulated miRNAs (hsa-miR-10401-3p, hsa-miR-548ak, hsa-miR-141-3p, and hsa-miR-3940-3p) exhibited the most substantial dysregulation, with log_2_FC greater than 1.0 (equivalent to FC > 2). A complete list of SDE-miRNAs, along with their associated statistics, is provided in Supplementary Data S5.

### 2.3. Target Gene Identification and Pathway Enrichment Analysis

**Figure 2:**
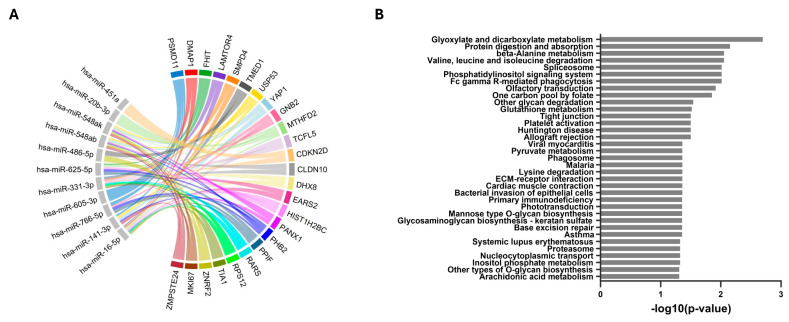
Functional role of the 15 SDE-miRNAs in plasma from PWH with cirrhosis one year after completion of HCV therapy. (**A**) A chord diagram illustrating the regulatory network between the SDE-miRNAs (left) and their top 25 predicted target genes (right). Significant miRNA-target interactions were defined by a false discovery rate (FDR) ≤ 0.05. (**B**) Bar plot of the significantly enriched Kyoto Encyclopedia of Genes and Genomes (KEGG) pathways identified from the SDE-miRNA target genes. Pathways are ranked by their enrichment *p*-value, with significance defined as *p* ≤ 0.05. **Abbreviations**: *PSMD11*, proteasome 26S subunit, non-ATPase 11; *DMAP1*, DNA methyltransferase 1 associated protein 1; *FHIT*, fragile histidine triad diadenosine triphosphatase; *LAMTOR4*, late endosomal/lysosomal adaptor, MAPK and MTOR activator 4; *SMPD4*, sphingomyelin phosphodiesterase 4; *TMED1*, transmembrane P24 trafficking protein 1; *USP53*, ubiquitin specific peptidase 53; *YAP1*, Yes1 associated transcriptional regulator; *GNB2*, G protein subunit beta 2; *MTHFD2*, methylenetetrahydrofolate dehydrogenase (NADP + dependent) 2; *TCFL5*, transcription factor like 5; *CDKN2D*, cyclin dependent kinase inhibitor 2D; *CLDN10*, claudin 10; *DHX8*, DEAH-box helicase 8; *EARS2*, glutamyl-tRNA synthetase 2; *HIST1H2BC*, H2B clustered histone 4; *PANX1*, pannexin 1; *PHB2*, prohibitin 2; *PPIF*, peptidylprolyl isomerase F; *RARS*, arginyl-tRNA synthetase 1; *RPS12*, ribosomal protein S12; *TIA1*, TIA1 cytotoxic granule associated RNA binding protein; *ZNRF2*, zinc and ring finger 2; *MKI67*, marker of proliferation Ki-67; *ZMPSTE24*, zinc metallopeptidase STE24.

miRNA-gene interaction analysis indicated that these 15 SDE miRNAs potentially regulate multiple genes, with the top 25 target genes shown in Figure 2A and Supplementary Data S6.

We identified 35 pathways significantly regulated by the 15 SDE miRNAs. Notably, the most prominent and highly enriched pathways were involved in amino acid metabolism, including protein digestion and absorption, valine, leucine, and isoleucine degradation, and beta-alanine metabolism (Figure 2B and Supplementary Data S7).

### 2.4. Correlation Between SDE miRNAs and Metabolites

**Figure 3:**
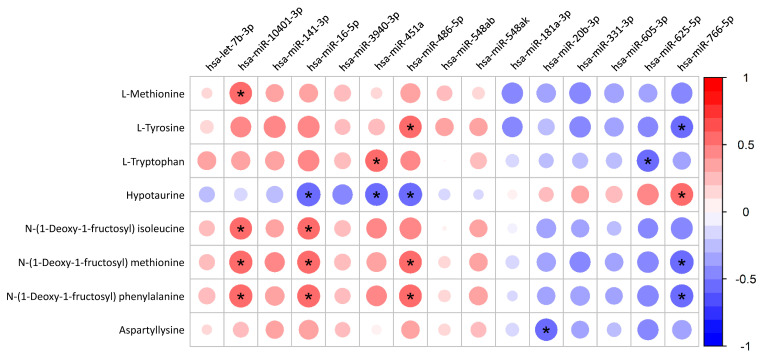
Spearman correlations between the expression of SDE-miRNAs and the plasma levels of metabolites. SDE miRNAs are on the horizontal axis and metabolites on the vertical axis. Colours represent the correlation direction (colour legends are shown on the right), and the size represents the strength of the correlation. An asterisk (*) denotes a statistically significant correlation, defined as a q-value ≤ 0.2. **Abbreviations:** SDE, significantly differentially expressed.

To experimentally investigate the functional impact of the SDE-miRNA signature, we correlated their expression levels with our untargeted plasma metabolomic data (Figure 3 and Supplementary Data S8). This analysis confirmed that the most significant and consistent associations were with plasma amino acids and their derivatives. Specifically, while the expression of upregulated miRNAs (e.g., hsa-miR-10401-3p, hsa-miR-16-5p) was positively correlated with plasma levels of L-methionine and several N-(1-Deoxy-1-fructosyl)-amino acid derivatives, the expression of downregulated miRNAs (e.g., hsa-miR-766-5p) showed a negative correlation.

The analysis also revealed other key correlations. Plasma hypotaurine was negatively correlated with upregulated miRNAs (hsa-miR-16-5p, hsa-miR-451a, and hsa-miR-486-5p) and positively correlated with the downregulated hsa-miR-766-5p. Additionally, the downregulated hsa-miR-20b-3p showed a significant negative correlation with plasma aspartyllysine.

## 3. Discussion

Our study provides a comprehensive characterization of the plasma miRNA profile in PWH with a history of HCV who presented cirrhosis one year after successful HCV treatment. A key finding is the identification of a distinct miRNA signature comprising 15 SDE miRNAs associated with the dysregulation of amino acid metabolism. Notably, this finding is strongly supported by the convergence of our multi-omic approach, where both miRNA profiling and our parallel metabolomic analyses independently highlighted the same altered metabolic pathways, thereby providing robust, cross-validated evidence for our results.

### 3.1. Pro-Fibrotic and Inflammatory miRNA Signature Despite SVR

Our data reveal a central theme: HCV eradication is insufficient to resolve the underlying pro-fibrotic and inflammatory signalling in patients with significant pre-existing liver stiffness. Instead, a distinct miRNA profile persists, reflecting the continued activity of molecular pathways that sustain liver injury.

Among the most relevant changes, hsa-miR-141 is upregulated, consistent with its role in activating hepatic stellate cells (HSC) [15], a critical event in the progression of liver fibrosis. This aligns with previous findings where hsa-miR-141 was found to be upregulated in fibrotic liver tissues [16], indicating that the molecular machinery driving fibrosis remains active in these patients. However, this miRNA appears to undergo a profound shift during HCC development, since in patients with HCC its expression is suppressed, in both serum [17] and tissue [18], suggesting a context-dependent role in which it may act as a potential suppressor of HCC development.

Furthermore, the upregulation of the hsa-miR-548 family, specifically hsa-miR-548ak, points towards a persistent inflammatory state. Previous studies have established an association between the hsa-miR-548 family expression levels and target genes involved in inflammatory pathways [19,20]. Specifically, this miRNA family regulates immune response in the context of viral infections, such as viral hepatitis [21]. Although no previous studies have investigated the specific role of hsa-miR-548ak in liver fibrosis, its continued high expression one year after of the completion of HCV therapy strongly suggests a state of low-grade residual inflammation that likely contributes to sustained liver injury and stiffness.

Other differentially expressed miRNAs also contribute to this unresolved profile. Elevated levels of hsa-miR-16-5p, commonly linked to cellular stress and injury [22], and hsa-miR-3940, related to cell cycle regulation and immune infiltration [23], suggest sustained hepatic damage. In contrast, increased hsa-let-7b-3p -despite the anti-inflammatory properties attributed to the let-7 family [24]-may represent a compensatory attempt to counteract inflammation. Similarly, decreased expression of hsa-miR-181a, hsa-miR-20b and hsa-miR-605-3p, previously linked to inflammatory and multimorbidity burden in other populations [25,26,27], could reflect an adaptive response aimed at limiting chronic inflammation in cirrhotic PWH.

Consistent with these observations, target prediction analysis revealed that the top 25 genes potentially regulated by the SDE miRNAs include critical regulators of cell proliferation and survival (*MKI67*, *YAP1*, *PPIF*) [28,29,30], and immune or inflammatory processes (*TIA1*, *SMPD4*) [31,32]. Their enrichment reinforces the notion that the altered miRNA profile in cirrhotic PWH may actively influence fibrosis, inflammation, and metabolic dysregulation even after HCV clearance.

### 3.2. Dysregulation of Amino Acid Metabolism Leads to Persistent Liver Disease

The most relevant finding of our study is the profound alteration in miRNAs that regulate, or are associated with, amino acid metabolism pathways. This aligns with a growing body of evidence indicating that impaired hepatic function leads to a systemic imbalance in amino acid homeostasis, which is closely linked to the severity of liver disease [33,34].

Supporting this, target prediction and pathway enrichment analyses highlighted that several of the top 25 predicted genes regulated by SDE miRNAs are directly involved in metabolic regulation, particularly amino acid–related pathways. Among them, *MTHFD2* plays a central role in one-carbon metabolism and has been reported to be required for cancer proliferation [35]. Likewise, *RARS* links arginine availability to protein translation and amino acid–sensing pathways [36]. Consistently, the most enriched pathways included, among others, glyoxylate and dicarboxylate metabolism, β-alanine metabolism, valine/leucine/isoleucine degradation, the one-carbon pool by folate, lysine degradation, among others. These results suggest that the altered miRNA profile in cirrhotic PWH may influence amino acid–related metabolic pathways, potentially contributing to liver dysfunction.

Consistent with these predictions, several dysregulated miRNAs showed significant correlations with plasma amino acids and their derivatives. We found negative correlations between the downregulated hsa-miR-766-5p and L-tyrosine, N-(1-Deoxy-1-fructosyl) methionine, and phenylalanine. In line with this, hsa-miR-10401-3p, the most highly upregulated miRNA in our cohort, was positively correlated with L-methionine and N-(1-Deoxy-1-fructosyl) amino acid derivatives. Similar positive correlations were observed for hsa-miR-16-5p and hsa-miR-486-5p. These findings support the potential link between these miRNAs and amino acid–related pathways, reflecting a complex dysregulation which may contribute to liver damage. In this setting, higher plasma levels of methionine, tyrosine, and phenylalanine have been consistently associated with the severity of liver cirrhosis [37,38,39], aligning with our findings.

Regarding tryptophan metabolism, its dysregulation is particularly crucial in liver fibrosis, as this is the primary organ for tryptophan catabolism [40,41]. Thus, the cirrhosis leads to a failure in this process, resulting in elevated plasma tryptophan levels, which are directly associated with the severity of the disease [42]. Our study identified an inverse correlation between elevated plasma tryptophan levels and the decreased expression of hsa-miR-625-5p in patients with cirrhosis. Although the exact role of this miRNA in tryptophan metabolism is not fully elucidated, it is known that hsa-miR-625 has been linked to metabolic processes [43], other infectious diseases [44], and several types of cancer [45,46].

Conversely, we observed a positive correlation between hsa-miR-766-5p and hypotaurine, a metabolite recognized for its antioxidant role and hepatoprotective functions [47]. This association suggests that reduced hsa-miR-766-5p levels may reflect a lower bioavailability of hypotaurine, potentially impairing a crucial protective mechanism against oxidative stress in the fibrotic liver. Consistent with this, we also found a negative correlation between hypotaurine levels and the expression of hsa-miR-16-5p, hsa-miR-451a, and hsa-miR-486-5p, further reinforcing the evidence of lower plasma levels of hypotaurine. Therefore, our findings suggest a failure in this protective and antioxidant mechanism in PWH with cirrhosis.

Overall, we found a pro-inflammatory miRNA signature that sustains fibrogenic processes and places a heavy burden on hepatic metabolic function, leading to the observed dysregulation in amino acid pathways. This metabolic imbalance, in turn, may further fuel inflammation and oxidative stress (e.g., through reduced hypotaurine bioavailability), creating a self-sustaining vicious cycle of injury and metabolic dysfunction that is not resolved by viral eradication alone.

From a pharmacological perspective, our findings open avenues for therapeutic innovation. The persistence of a pro-fibrotic and inflammatory miRNA signature despite HCV cure suggests that antiviral therapy alone is insufficient to restore hepatic homeostasis. Several of the dysregulated miRNAs identified (e.g., hsa-miR-16-5p) are involved in pathways targeted by antifibrotic and anti-inflammatory drugs [48] indicating potential synergy between molecular profiling and pharmacotherapy. Moreover, the strong association between miRNA expression and amino acid metabolism highlights the possibility of integrating metabolic modulators—such as agents influencing methionine or tryptophan pathways—into post-SVR management. These insights support the concept of miRNA-based biomarkers as tools for personalized medicine, guiding adjunctive therapies aimed at mitigating residual liver injury in PWH.

### 3.3. Strengths and Limitations

The primary strength of this study is its robust, integrated multi-omic design. By combining miRNA profiling with untargeted metabolomics, we not only identified a miRNA signature of cirrhosis but also provided experimental validation of its functional link to amino acid metabolism. This was performed in a well-characterized clinical cohort of PWH post-HCV cure, a clinically critical but understudied population.

However, several limitations must be acknowledged. First, the sample size is modest, which may limit the statistical power to detect miRNAs with more subtle expression changes. To address this, we employed a conservative analytical pipeline including DESeq2 for stable variance estimation and FDR correction to minimize false positives. Furthermore, the functional relevance of the identified signature was cross-validated through correlation with plasma metabolites, reinforcing the reliability of the findings despite the sample size constraints. Second, the cross-sectional design at a single time point (one year after HCV therapy) precludes inference on causality or the temporal dynamics of the molecular profiles. Longitudinal studies in larger, multicenter cohorts are needed to confirm the clinical utility of the identified miRNA signatures. Third, the relatively narrow age range and the exclusive Caucasian ethnicity of the study population reflect the specific demographic characteristics of PWH infected with HCV in our region. This homogeneity limits the generalizability of our findings to other age groups or ancestries. Fourth, only a subset of the initial eligible cohort was available for evaluation one year after HCV therapy, due to unavailability of LSM data or lack of available omic datasets, which may have influenced the representativeness of the study population. Fifth, metabolomic analyses were performed exclusively in positive-ion mode to prioritize the detection of amino acids and amines. Consequently, the coverage of metabolite classes that ionize preferentially in negative mode (e.g., organic acids, lipids) may be limited. Finally, our analysis demonstrates an association, and thus, further functional studies are required to confirm the precise regulatory mechanisms. Moreover, since HIV infection induces persistent immune activation and dysregulation, it remains unclear whether the miRNA signature is unique or exacerbated in the PWH population. Future comparative studies of cirrhotic HCV versus HIV/HCV infected cohorts are needed to clarify it. Additionally, the lack of a control group consisting of PWH without a history of HCV infection prevents us from determining whether the miRNA profiles of non-cirrhotic patients fully normalize to a baseline state (non-HCV-infected liver). Future comparative studies are needed to address this question.

## 4. Materials and Methods

### 4.1. Study Population

A cross-sectional study was conducted among PWH coinfected with HCV who had advanced fibrosis or cirrhosis and successfully cleared HCV infection following interferon (IFN)-based therapy (pegylated interferon α [peg-IFN-α] with ribavirin, with or without direct-acting antivirals [DAAs]) or IFN-free DAA therapy between 2012 and 2017 across 10 centres in Spain (see Appendix A). SVR was defined as undetectable HCV-RNA 12–24 weeks after completion of anti-HCV treatment depending on the regimen, and all patients maintained SVR throughout the follow-up period.

Key inclusion criteria included stable antiretroviral therapy (ART) for at least six months, and plasma HIV RNA < 50 copies/mL. Exclusion criteria included HCV reinfection, hepatitis B virus (HBV) co-infection, acute hepatitis C, hepatocellular carcinoma (HCC), and hepatic decompensation.

The study protocol was approved by the Research Ethics Committee of the Institute of Health Carlos III (CEI PI 72_2021) and was conducted in accordance with the Declaration of Helsinki. All participants provided written informed consent before enrollment.

### 4.2. Clinical Data and Sample Collection

Peripheral blood samples were collected in EDTA tubes approximately one year after the completion of HCV therapy. Plasma was isolated by Ficoll-Paque density gradient centrifugation and subsequently stored at −80 °C in the Spanish HIV HGM Biobank until analysis. Corresponding epidemiological and clinical data were retrospectively collected from a confidential online form and cross-verified against patient medical records.

### 4.3. Outcome Variable

The primary outcome was cirrhosis status at the time of sampling, approximately one year after the completion of HCV therapy. We defined cirrhosis as a liver stiffness measurement (LSM) ≥ 12.5 kPa. LSM was measured by transient elastography using a FibroScan^®^ (Echosens, Paris, France), as previously described [49]. The selection of this threshold for cirrhosis is supported by its widespread acceptance in the literature [50,51].

### 4.4. RNA Extraction and miRNome High Throughput Sequencing

We used miRNeasy Serum/Plasma Advanced Kit (Qiagen, Venlo, The Netherlands) to extract total RNA from plasma, which enriched in small RNAs following the manufacturer’s instructions. Sequencing libraries were prepared using the NEXTFLEX Small RNA-Seq Kit v4 for Illumina Platforms (ref. NOVA-5132, PerkinElmer, Waltham, MA, USA) according to the manufacturer’s protocol. Libraries were sequenced on an Illumina NextSeq 2000 (Illumina, Inc., San Diego, CA, USA) instrument across four runs, generating 1 × 51 bp single-end reads with dual 10 bp index reads. This strategy was designed to achieve a minimum of 10 million reads per sample (detailed information is provided in Supplementary Data S1).

### 4.5. Bioinformatics Analysis

We analyzed raw data using a specific bioinformatic pipeline detailed in Supplementary Data S2. The workflow consisted of the following steps: (i) raw reads were assessed for quality with FastQC (v.0.11.9-JAVA-11) [52]; (ii) adapter sequences were trimmed using cutadapt (v.4.0) [53]; and (iii) the processed reads were aligned to the human genome (GRCh38) and quantified against known miRNA precursors from miRBase (v22.1) using miRDeep2 [54,55].

### 4.6. Metabolites Detection and Identification

Untargeted metabolomic analysis was performed at the Centro de Metabolómica y Bioanálisis (CEMBIO, Madrid, Spain). A detailed description of the methodology, including metabolite extraction, quality control (QC) procedures, analytical conditions, and reagent lists, is available in Supplementary Data S3.

Briefly, plasma samples were deproteinized, dried, and subsequently analyzed by capillary electrophoresis-mass spectrometry (CE-MS). QC samples, prepared by pooling equal volumes of plasma, were processed alongside experimental samples. An Agilent 6224 TOF-MS system (Agilent Technologies, Santa Clara, CA, USA) was used for analysis, and data acquisition was performed using MassHunter Workstation software vB.09.00 (Agilent Technologies, Waldrobonn, Germany). Raw data files were processed with Profinder software B.10.0.2 (Agilent Technologies, Santa Clara, CA, USA) for feature alignment and integration.

### 4.7. Statistical Analysis

The statistical analysis was performed using the R statistical software (v4.3.1).

Differences in clinical and demographic characteristics between the cirrhosis and non-cirrhosis groups were assessed using the Mann–Whitney U test for continuous variables and the Chi-squared test for categorical variables.

Prior to differential expression analysis, we performed principal component analysis (PCA) to explore potential batch effects across sequencing runs. We employed DESeq2 method (v1.42.1) to normalize miRNA counts. Later, a generalized linear model (GLM) with a negative binomial distribution was performed to analyze differential expression between PWH with and without cirrhosis one-year after successful completion of HCV therapy. Relevant covariates were incorporated into each model through a forward stepwise procedure based on the lowest Akaike information criterion (AIC). Adjustment factors included age, gender and type of HCV therapy. Significantly differentially expressed (SDE) miRNAs were defined by an absolute log_2_ fold change (|log_2_FC|) > 0.585 and a false discovery rate (FDR) by Benjamini–Hochberg adjusted *p*-value (q-value) ≤ 0.2.

We then investigated the correlations between expression of SDE-miRNAs and plasma metabolite levels using Spearman’s correlation. Correlations were considered statistically significant at a *p*-value < 0.05 and a q-value < 0.2.

### 4.8. Target Gene Identification and Pathway Enrichment Analysis

We performed target gene and pathway enrichment analyses to elucidate the biological function of the SDE-miRNAs. First, SDE miRNA-target interactions were assessed with the CORALIS R package 1.0.0 [56], considering only experimentally confirmed interactions. Target genes were limited to those with at least two interactions with SDE miRNAs. Significant interactions were defined as those with an FDR-adjusted *p*-value ≤ 0.05. Second, MiEAA webtool (https://ccb-compute2.cs.uni-saarland.de/mieaa/ (accessed on 15 October 2025)) [57] was used to carry out functional enrichment analysis of SDE miRNAs using the Kyoto Encyclopedia of Genes and Genomes (KEGG) database. Significant pathways were defined as those with a *p*-value ≤ 0.05.

## 5. Conclusions

In conclusion, our findings indicated that PWH with cirrhosis, exhibit a dysregulation of their plasma miRNAs profile, even one year after successful HCV eradication. This altered signature is strongly associated with shifts in amino acid metabolism, a finding corroborated by metabolomic data. These results provide clear evidence that HCV cure does not equate to hepatic metabolic recovery. Therefore, these alterations after HCV eradication highlight the need for continued, long-term exhaustive monitoring of this population due to a potential ongoing risk of liver disease progression.

## Figures and Tables

**Table 1:** Epidemiological and clinical characteristics of people with HIV one year after completion of HCV therapy, stratified by cirrhosis status.

	All Patients	Non-Cirrhosis	Cirrhosis	*p*-Value
No.	47	15	32	
Age (years)	52 (51–55)	54 (52–56)	52 (50–55)	0.315
Gender (male)	37 (78.7%)	12 (80.0%)	25 (78.1%)	0.999
BMI (kg/m^2^)	24.7 (21.4–27.2)	24.1 (21.2–26.2)	24.9 (21.7–27.6)	0.477
Smoker				0.752
Never	5 (11.4%)	2 (13.3%)	3 (10.3%)	
Previous (>6 months)	15 (34.1%)	4 (26.7%)	11 (37.9%)	
Current	24 (54.5%)	9 (60.0%)	15 (51.7)	
Alcohol intake (>50 g/day) (*n* = 46)				0.282
Never	25 (55.6%)	10 (66.7%)	15 (50%)	
Previous (>6 months)	16 (35.6%)	5 (33.3%)	11 (36.7%)	
Current	4 (8.9%)	-	4 (13.3%)	
Persons who inject drugs				0.378
Never	13 (28.3%)	6 (40%)	7 (22.6%)	
Previous (>6 months)	33 (71.7%)	9 (60%)	24 (77.4%)	
Current	-			
**Liver markers**				
LSM (kPa)	14.3 (9.0–21.9)	7.2 (6.0–8.5)	18.6 (14.3–27.9)	**<0.001**
HSI	32.1 (29.0–35.4)	31.8 (30.0–34.3)	32.1 (28.5–36.9)	0.989
APRI	0.50 (0.35–0.69)	0.41 (0.28–0.46)	0.58 (0.42–0.83)	0.054
**HCV markers**				
HCV genotype				0.288
1	34 (72.4%)	11 (73.3%)	22 (71.0%)	
3	8 (17.0%)	4 (26.7%)	4 (12.9%)	
4	5 (10.6%)	-	5 (16.1%)	
**HCV therapy**				**<0.001**
pegIFN	27 (57.4%)	14 (93.3%)	13 (40.6%)	
DAAs	20 (42.6%)	1 (6.7%)	19 (59.4%)	
**HIV markers**				
Previous AIDS	1 (2.2%)	1 (6.7%)	0	0.708
CD4+ T-cells/mm^3^	522.5 (279.0–714.3)	536.0 (348.0–710.5)	487.0 (273.3–214.3)	0.705
CD4+ T-cells < 500 cells/mm^3^	23 (47.9%)	6 (37.5%)	17 (53.1%)	0.475
**ART**				0.373
NRTI + NNRTI	17 (37.8%)	6 (42.9%)	11 (35.5%)	
NRTI + II	18 (37.8%)	3 (21.4%)	14 (45.2%)	
NRTI + PI	7 (15.6%)	4 (28.6%)	3 (9.7%)	
PI + II + NNRTI/MVC	1 (2.2%)	-	1 (3.2%)	
Others	3 (6.7%)	1 (7.1%)	2 (6.5%)	

Data are presented as median (interquartile range; IQR) or n (%). *p*-values were calculated using the Mann–Whitney U test for continuous variables and the Chi-squared test for categorical variables. HIV-related markers show the patient’s condition before starting HCV therapy. Data were not available for all patients for the following variables: smoking status (*n* = 45), alcohol intake (*n* = 46), persons who inject drugs (*n* = 46), HSI (*n* = 37), APRI (*n* = 37), previous AIDS diagnosis (*n* = 38), and antiretroviral therapy (ART) status (*n* = 46). Bold text indicates variable group headings and statistically significant *p*-values. **Abbreviations**: HCV, hepatitis C virus; HIV, human immunodeficiency virus; BMI, body mass index; LSM, liver stiffness measurement; kPa, kilopascal; HSI, hepatic steatosis index; pegIFN, pegylated interferon; DAAs, direct-acting antivirals; AIDS, acquired immune deficiency syndrome; ART, antiretroviral therapy; NRTI, nucleoside analogue HIV reverse transcriptase inhibitor; NNRTI, non-nucleoside analogue HIV reverse transcriptase inhibitor; II, HIV integrase inhibitor; PI, HIV protease inhibitor.

## Data Availability

Raw sequences of miRNAs data are available in BioStudies repository (accession code S-BSST2284) (https://www.ebi.ac.uk/biostudies/studies/S-BSST2284?key=d280f317-ddea-4861-aa1e-51e56c8db71c (accessed on 13 January 2026)).

## Supplementary Materials

The following supporting information can be downloaded at: https://www.mdpi.com/article/10.3390/ph19010170/s1. Supplementary Data S1: Extended material and methods. Supplementary Data S2: Extension of bioinformatic pipeline. Supplementary Data S3: Extension of metabolite detection and annotation. Supplementary Data S4: Principal component analysis of miRNAs sequencing runs from all samples included in the study. Supplementary Data S5: Differential expression analysis of miRNAs between people with HIV (PWH) with or without clinically relevant liver stiffness (LSM ≥ 12.5 kPa) at one year after completion of HCV therapy. Supplementary Data S6: Top 25 target genes regulated by the 15 SDE miRNAs in people with HIV (PWH) with cirrhosis (LSM ≥ 12.5 kPa) one year after completion of HCV therapy. Supplementary Data S7: Enrichment and annotation pathways analysis of differentially expressed miRNAs between people with HIV (PWH) with or without clinically relevant liver stiffness (LSM ≥ 12.5 kPa) at one year after completion of HCV therapy. Supplementary Data S8: Correlations between expression of SDE miRNAs with plasma metabolite levels in PWH at one year after completion of HCV therapy. References [52,53,54,55,58,59,60,61] are cited in the Supplementary Material.

## Abbreviations

ART	Antiretroviral therapy
BMI	Body mass index
CE-MS	Capillary electrophoresis-mass spectrometry
DAA	Direct-acting antiviral
FC	Fold Change
FDR	False discovery rate
GLM	Generalized linear models
HBV	Hepatitis B virus
HCC	Hepatocarcinoma
HCV	Hepatitis C virus
HIV	Human Immunodeficiency Virus
HSC	Hepatic stellate cells
HSI	Hepatic steatosis index
IFN	Interferon
KEGG	Kyoto Encyclopedia of Genes and Genomes
LSM	Liver stiffness measurement
*MKI67*	Marker of proliferation Ki-67
miRNAs	microRNAs
mRNA	Messenger RNA
*MTHFD2*	Methylenetetrahydrofolate dehydrogenase (NADP + dependent) 2
PCA	Principal component analysis
*PPIF*	Peptidylprolyl isomerase F
PWH	People with HIV
*RARS*	Arginyl-tRNA synthetase
SDE	Significantly differentially expressed
SMPD4	Sphingomyelin phosphodiesterase 4
SVR	Sustained Virologic response
*TIA1*	TIA1 cytotoxic granule associated RNA binding protein
*YAP1*	Yes1 associated transcriptional regulator

## Appendix A

Members of the Marathon Study Group

**Hospital General Universitario Gregorio Marañón, Madrid:** A Carrero, P Miralles, JC López, F Parras, B Padilla, T Aldamiz-Echevarría, F Tejerina, C Díez, L Pérez-Latorre, C Fanciulli, I Gutiérrez, M Ramírez, S Carretero, JM Bellón, J Bermejo, and J Berenguer.

**Hospital Universitario La Paz, Madrid:** V Hontañón, JR Arribas, ML Montes, I Bernardino, JF Pascual, F Zamora, JM Peña, F Arnalich, M Díaz, J González-García.

**Hospital Universitari Vall d’Hebron, Barcelona:** E Van den Eynde, M Pérez, E Ribera, M Crespo.

Hospital Universitario Príncipe de Asturias, Alcalá de Henares: A Arranz, E Casas, J de Miguel, S Schroeder, J Sanz.

**Hospital Donostia, San Sebastián:** MJ Bustinduy, JA Iribarren, F Rodríguez-Arrondo, MA Von-Wichmann.

Hospital Universitario de La Princesa, Madrid: J Sanz, I Santos.

Hospital Clínico San Carlos, Madrid: J Vergas, MJ Téllez.

Hospital Clínico Universitario, Valencia: A Ferrer, MJ Galindo.

**Hospital Universitario Ramón y Cajal, Madrid:** JL Casado, F Dronda, A Moreno, MJ Pérez-Elías, MA Sanfrutos, S Moreno, C Quereda.

Hospital General Universitario, Valencia: L Ortiz, E Ortega.

**Fundación SEIMC-GESIDA, Madrid:** M Yllescas, P Crespo, E Aznar, H Esteban.
