# Supplementary material for: Plasma miRNA-Metabolite Dysregulation in People with HIV with Cirrhosis Despite Successful HCV Cure
Source: Pharmaceuticals (Basel). 2026 Jan 19;19(1):170. doi: 10.3390/ph19010170 (PMC12845460; doi:10.3390/ph19010170)
Supplement: Supplementary file 1 [file pharmaceuticals-19-00170-s001.zip › pharmaceuticals-4073305-supplementary.pdf]

Title page

Title: Plasma miRNA-metabolite dysregulation in people with HIV with cirrhosis despite successful HCV cure

SUPPLEMENTARY DATA INDEX

Supplementary Data S1: Extended material and methods. 2

Supplementary Data S2: Extension of bioinformatic pipeline. 2

Supplementary Data S3: Extension of metabolite detection and annotation. 3

Supplementary Data S4: Principal component analysis of miRNAs sequencing runs from all samples included in the study. 7

Supplementary Data S5: Differential expression analysis of miRNAs between people with HIV (PWH) with or without clinically relevant liver stiffness (LSM≥12.5kPa) at one year after completion of HCV therapy. 8

Supplementary Data S6: Top 25 target genes regulated by the 15 SDE miRNAs in people with HIV (PWH) with cirrhosis (LSM≥12.5kPa) one year after completion of HCV therapy. 9

Supplementary Data S7: Enrichment and annotation pathways analysis of differential expression miRNAs between people with HIV (PWH) with or without clinically relevant liver stiffness (LSM≥12.5kPa) at one year after completion of HCV therapy. 11

Supplementary Data S8: Correlations between expression of SDE miRNAs with metabolites plasma levels in PWH at one year after completion of HCV therapy. 13

Supplementary Data S1. Extended material and methods.

Briefly, the protocol comprised ligation of both 3' and 5' adapters to the RNA molecules, reverse transcription to generate cDNA, and amplification and indexing by PCR of cDNA molecules that had adapters on both ends. PCR was performed using the UDI barcoded primer mix included in the kit, so the final libraries had unique dual indexes. All purification steps were performed using the cleanup beads included in the kit.

Final libraries were analyzed using Agilent Bioanalyzer HS DNA Assay (ref. 5067-4626) and quantified by qPCR using the KAPA Library Quantification Kit KK4835 (ref. 07960204001, Roche). Libraries were sequenced 1 * 51+10+10 bp on Illumina's NextSeq2000.

Supplementary Data S2. Extension of the bioinformatic pipeline.

Bcl2fastq (Illumina) was used to convert sequence data from BCL files to FASTA. Each read was attributed to the appropriate sample once the samples were demultiplexed. A particular bioinformatic pipeline was then used to evaluate the raw data in order to identify and quantify known miRNAs.

Initially, reads with uncertain base calls that did not satisfy the Illumina chastity filter based on quality criteria were filtered out. FastQC (v.0.11.9-JAVA-11) (1) was used to assess the quality of the remaining reads, and cutadapt (v.4.0) was used to trim adapter sequences (2). MiRDeep2 was then used to process the reads (3). This software uses the mapper.pl module based on Bowtie1 to align the reads to the reference human genome (GRCh38). The only alignments kept were those that mapped to no more than five distinct loci in the genome and those that had zero mismatches in the seed region.

Quantifier.pl module was used to quantify miRNAs, which determines the expression of the corresponding known miRNAs in two steps. First, predefined mature miRNA sequences were mapped against the predefined precursors in miRBase (v22.1) (4), the public repository for all reported miRNA sequences. Second, sequencing reads were mapped against the precursor sequences. For quantification, reads falling into an interval of two nucleotides upstream and five nucleotides downstream of the mature miRNA sequences were determined.

Supplementary Data S3. Extension of metabolite detection and annotation.

1. ***Reagents and standards***

LC-MS grade methanol (MeOH), acetonitrile (ACN), and isopropanol (IPA) were obtained from Fisher Scientific (Pennsylvania, United States). Analytical grade ammonia solution (28%, GPR RECTAPUR®) and acetic acid glacial (AnalaR® NORMAPUR®) were obtained from VWR Chemicals (Pennsylvania, United States). The ammonium fluoride (NH4F) (ACS reagent, ≥ 98%) and Methyl-tert-butyl ether (MTBE) were purchased from Sigma-Aldrich (Steinheim, Germany). Reverse-osmosed ultrapure water for aqueous solutions was obtained from a Milli-Qplus185 system (Millipore, Billerica, MA, USA). For analytical quality assurance, C17–Sphinganine and d_{31}–palmitic acid were purchased from Avanti Polar Lipids (Alabaster, Alabama, USA) for LC-MS. In the same way, for CE-MS quality assurance, methionine sulfone, paracetamol, 2-(N-morpholino) ethanesulfonic acid (MES), and formic acid were acquired from Sigma (Steinheim, Germany). Reference mass solutions for LC-MS and CE-MS were obtained from Agilent Technologies.

2. ***Blood samples***

Peripheral blood samples were obtained in EDTA tubes. Plasma fraction was obtained after Ficoll-Paque density gradient centrifugation and subsequently stored at the Spanish HIV HGM Biobank at -80ºC.

3. ***Viral inactivation***

Viral inactivation of plasma samples was performed by mixing 300 µL of plasma with 900 µL of cold MeOH:EtOH (1:1, v/v), reaching a 1:3, v/v ratio. After that, the samples were vortex mixed for 1 min and introduced on an ice-bath for 5 min. Finally, the samples were centrifuged at 16000g, during 20 min and 4ºC, and then they were stored at -80ºC until analysis.

4. ***Capillary Electrophoresis Analysis***

   4.1. Metabolites extraction

Inactivated plasma samples were also prepared at the "Centro de Metabolómica y Bioanálisis, CEMBIO" (Madrid, Spain). The samples were thawed on ice and vortex-mixed for 2 min. The samples were centrifuged for 15 min at 4ºC and 16,000xg, and 400 µL of the supernatant was transferred to an Eppendorf tube. Next, the samples were dried on the SpeedVac Concentrator (Thermo Fisher Scientific, Waltham, MA, USA) and resuspended in 200 µL of 0.1M Formic acid containing 0.2 mM methionine sulfone, 1 mM paracetamol, 0.25 mM (MES) and 25% ACN as internal standards (IS). Samples were then vortex-mixed for 1 min and transferred to a 30-kDa protein cut-off filter for the deproteinization process through a centrifugation step using a Centrifree ultracentrifugation device (Millipore Ireland Ltd., Cork,

Ireland) for 90 min at 2000xg and 4ºC. The filtrate was transferred to a chromacol vial for CE-MS analysis. The vials were then centrifuged for 10 min at 2000xg and 4ºC.

4.2. Quality Management Assurance and Blank samples

Equal volumes of each plasma sample (50 µL) were pooled into an Eppendorf tube to create Quality Control (QC) samples. QC samples were then prepared and processed in parallel with the rest of the plasma samples, following the metabolites extraction protocol described above. The system's stability, performance, and sample treatment method repeatability were all monitored during the run. Additionally, two blank samples were prepared along with the other samples using the same metabolite extraction procedure with the sample solvents. As was explained before, the blank samples were analyzed at the beginning and end of the analytical sequence to find potential contaminations.

4.3. Analytical conditions selected for a Metabolomics Analysis

An untargeted metabolomics-based approach was employed to comprehensively capture the diverse spectrum of plasma metabolites. Samples were analyzed using an Agilent CE 7100 coupled with an Agilent 6224 time-of-flight Mass Spectrometer (TOF-MS) analyzer. The coupling was performed with an electrospray source, helped by a sheath liquid, which consisted of methanol/water (1/1, v/v), formic acid (1 mM), and two reference masses (purine, m/z 121.050873; HP-0921, m/z 922.009798). The auxiliary sheath liquid, supplied by an Agilent 1200 ISO Pump, was used to compensate the volume and increase the necessary volatility for MS. For CE-MS separation analysis in positive ionization mode, a fused-silica capillary (Agilent Technologies; total length 100 cm x 50 µm i.d. x 360 µm) was used. First of all, a background electrolyte (BGE) (1 M formic acid in 10% methanol solution) solution was flushed for 5 min (950 mbar) through the capillary to condition and rinse it. A voltage of 30 kV was applied to displace the BGE ions during 10 s. Samples were then injected over 50s at 50mbar, followed by the BGE injection for 10 s at 100 mbar to improve the reproducibility of the analysis. The separation was carried out at a pressure of 25 mbar and a voltage of +30kV, in positive ionization mode, with an observed current of 24 µA under these conditions.

When the metabolites leave the capillary, a positively charger spray is formed with a flow rate of 0.6 mL/min (1:100) of the auxiliary liquid at 10 spig nebulization pressure with nitrogen and 3500 V of capillary voltage. The spray was dried with a hot nitrogen flow of 10 mL/min at 200 °C. The ions formed were directed to the TOF by 125 V - for adducts, dimmers and transformation data - and 200 V - for the acquisition of in-source fragment ions - fragmentor voltages, skimmer (65 V) and octopole (750 V). The MS was operated in ESI (+) positive polarity mode and a full range mass from 50 and 1050 Da was acquired at a scanning rate of 1.02 scans/s. Finally, selected samples were analyzed at the end of the analytical run

by applying a higher fragmentor voltage (125,150,175 and 200 V) to acquire in-source fragment ions (pseudo-MS/MS). By adopting this approach, the obtained spectrum is similar to a 10 eV MS/MS spectrum, although some fragment ions could differ (5, 6). The total time of the analytical run was 35 min. The CE-MS system was controlled by the MassHunter Workstation 6200 series TOF version B.09.00 c(B9044.0) acquisition software.

4.4. Metabolite Annotation process

Metabolite annotations were conducted utilizing a predefined CE-MS database meticulously tailored for CE-MS (5). This comprehensive in-house database is integrated into the CEU Mass Mediator (CMM) (7), an open-access resource, and was imported into the Agilent MassHunter Profinder software (B.10.0.2, Agilent Technologies, Santa Clara, CA, USA) for CE-MS data reprocessing.

5. Data Reprocessing

The raw data files were reprocessed by performing the hybrid strategy (8), which consisted of using an extensive compound database, metabolomics data was imported into the Agilent MassHunter Profinder software (B.10.0.2, Agilent Technologies, Santa Clara, CA, USA) to perform the time alignment and feature extraction using the "Batch Targeted Feature Extraction" mode. This strategy allowed us to ascertain the differences in the plasma metabolomic fingerprinting. The migration time (MT), monoisotopic exact mass, and molecular formula were all included in the database. The clustering of coeluting ions connected by charge-state, isotopic distribution, and/or the presence of various adducts and dimers in the analyzed samples was used for the feature-building process. The following adducts were selected positive CE-MS ionization mode, $[M+H]+$ and $[M+Na]+$ were selected. Common organic molecules (no halogens) for CE-ESI(+)-MS were used as the isotope grouping pattern, and the charge state was restricted to 1 - 2. We established the masses match tolerance at ± 20 ppm and ± 0.5 min for the RT. The RT score accounted for 100.00% of the total score. To complete the integration, we selected the "Agile 2” algorithm. The data format chromatogram employed was the centroid. The peak spectrum parameters were as follows: average scans > 10% of peak height, TOF spectra were excluded if above 20.0 % of saturation in the m/z ranges used, and empty spectrum will never return. The mass spectral data type was centroid. As a final post-processing filter, we only considered the features present in at least 50% of the samples within a single sample group.

5.1. Data normalization and filtering

For CE-MS metabolite analysis, normalization was achieved using the methionine sulfone IS, added during the initial sample preparation phase. Subsequently, metabolites were selected based on their coefficient of variation (CV) within the QC samples, applying a threshold of 30%.

REFERENCES

1. Bioinformatics B. FastQC a quality control tool for high throughput sequence data 2016. *Version 011.* 2017;5.
2. Martin M. Cutadapt removes adapter sequences from high-throughput sequencing reads. *EMBnet journal.* 2011;17(1):10-2.
3. Friedländer MR, Mackowiak SD, Li N, Chen W, and Rajewsky N. miRDeep2 accurately identifies known and hundreds of novel microRNA genes in seven animal clades. *Nucleic acids research.* 2012;40(1):37-52.
4. Kozomara A, and Griffiths-Jones S. miRBase: annotating high confidence microRNAs using deep sequencing data. *Nucleic acids research.* 2014;42(D1):D68-D73.
5. Mamani-Huanca M, de la Fuente AG, Otero A, Gradillas A, Godzien J, Barbas C, et al. Enhancing confidence of metabolite annotation in Capillary Electrophoresis-Mass Spectrometry untargeted metabolomics with relative migration time and in-source fragmentation. *Journal of chromatography A.* 2021;1635:461758.
6. Mamani-Huanca M, Gradillas A, Gil de la Fuente A, López-Gonzálvez An, and Barbas C. Unveiling the fragmentation mechanisms of modified amino acids as the key for their targeted identification. *Analytical Chemistry.* 2020;92(7):4848-57.
7. Gil-de-la-Fuente A, Godzien J, Saugar S, Garcia-Carmona R, Badran H, Wishart DS, et al. CEU Mass Mediator 3.0: A Metabolite Annotation Tool. *J Proteome Res.* 2019;18(2):797-802.
8. Fernández Requena B, Nadeem S, Reddy VP, Naidoo V, Glasgow JN, Steyn AJ, et al. LiLA: lipid lung-based ATLAS built through a comprehensive workflow designed for an accurate lipid annotation. *Communications Biology.* 2024;7(1):45.

Supplementary Data S4. Principal component analysis of miRNAs sequencing runs from all samples included in the study.

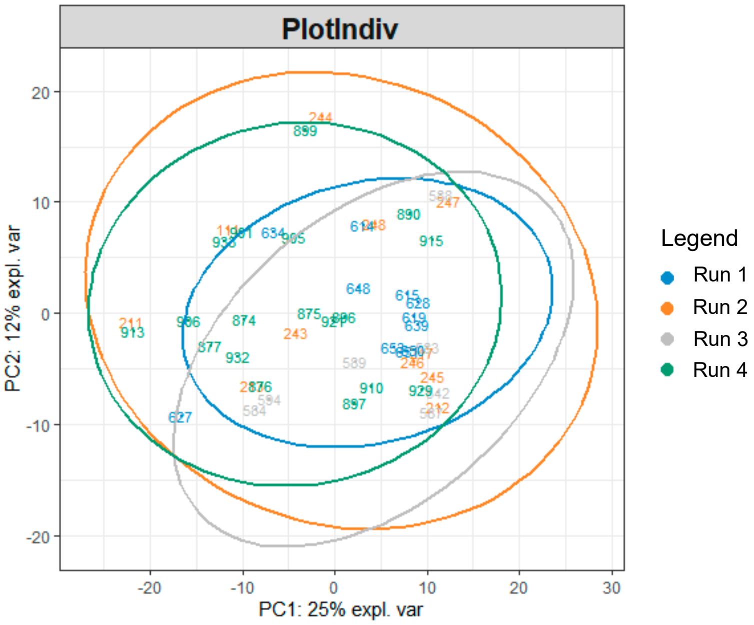

Supplementary Data S5. Significantly differentially expressed miRNAs between people with HIV (PWH) with or without cirrhosis (LSM≥12.5kPa) one year after completion of HCV therapy. **Statistics:** Generalized linear models (GLM) with negative binomial distribution adjusted by epidemiological characteristics (age, gender, HCV treatment) using a forward stepwise method. *P*-values were adjusted by FDR correction for multiple comparisons (Benjamini and Hochberg).

miRNAs	FC	Log2FC	p	q
hsa-let-7b-3p	1.743	0.801	**0.017**	**0.198**
hsa-miR-10401-3p	5.496	2.458	**0.001**	**0.027**
hsa-miR-10527-5p	1.440	0.526	**0.017**	**0.198**
hsa-miR-106b-3p	1.349	0.432	**0.007**	**0.101**
hsa-miR-12136	0.786	-0.348	**<0.001**	**<0.001**
hsa-miR-1226-3p	1.185	0.245	**0.001**	**0.021**
hsa-miR-1271-5p	1.128	0.174	**<0.001**	**0.008**
hsa-miR-141-3p	2.398	1.262	**0.002**	**0.029**
hsa-miR-155-5p	0.737	-0.441	**<0.001**	**<0.001**
hsa-miR-16-5p	1.526	0.610	**0.001**	**0.023**
hsa-miR-181a-3p	0.639	-0.646	**<0.001**	**<0.001**
hsa-miR-18b-5p	1.414	0.499	**0.012**	**0.155**
hsa-miR-20b-3p	0.482	-1.052	**<0.001**	**<0.001**
hsa-miR-22-3p	1.405	0.490	**0.011**	**0.150**
hsa-miR-3177-3p	0.853	-0.229	**<0.001**	**<0.001**
hsa-miR-323a-3p	0.689	-0.536	**<0.001**	**<0.001**
hsa-miR-331-5p	1.309	0.389	**<0.001**	**<0.001**
hsa-miR-331-3p	0.617	-0.698	**<0.001**	**<0.001**
hsa-miR-3940-3p	2.020	1.014	**<0.001**	**<0.001**
hsa-miR-451a	1.834	0.875	**0.008**	**0.114**
hsa-miR-486-5p	1.690	0.757	**0.015**	**0.187**
hsa-miR-500a-5p	1.231	0.299	**<0.001**	**<0.001**
hsa-miR-500b-5p	1.397	0.482	**<0.001**	**<0.001**
hsa-miR-501-5p	0.891	-0.166	**0.005**	**0.082**
hsa-miR-548ab	1.905	0.929	**0.012**	**0.155**
hsa-miR-548ak	2.524	1.336	**0.012**	**0.155**
hsa-miR-590-5p	1.204	0.268	**<0.001**	**0.011**
hsa-miR-605-3p	0.634	-0.659	**<0.001**	**<0.001**
hsa-miR-625-5p	0.599	-0.740	**0.004**	**0.073**
hsa-miR-6815-5p	1.258	0.331	**<0.001**	**<0.001**
hsa-miR-766-5p	0.643	-0.638	**<0.001**	**<0.001**
hsa-miR-874-3p	0.728	-0.458	**<0.001**	**<0.001**
hsa-miR-877-5p	0.876	-0.191	**<0.001**	**<0.001**

Supplementary Data S6. Top 25 target genes regulated by the 15 SDE miRNAs in people with HIV (PWH) with cirrhosis (LSM≥12.5kPa) one year after completion of HCV therapy.

Target Genes	p-value	FDR	miRNAs
PSMD11	**<0.001**	**0.006**	hsa-miR-16-5p / hsa-miR-141-3p / hsa-miR-766-5p / hsa-miR-605-3p / hsa-miR-331-3p
DMAP1	**<0.001**	**0.007**	hsa-miR-16-5p / hsa-miR-331-3p
FHIT	**<0.001**	**0.011**	hsa-miR-331-3p / hsa-miR-625-5p
LAMTOR4	**<0.001**	**0.011**	hsa-miR-16-5p / hsa-miR-331-3p
SMPD4	**<0.001**	**0.011**	hsa-miR-16-5p / hsa-miR-331-3p
TMED1	**<0.001**	**0.011**	hsa-miR-486-5p / hsa-miR-16-5p
USP53	**<0.001**	**0.011**	hsa-miR-16-5p / hsa-miR-548ab / hsa-miR-548ak / hsa-miR-141-3p
YAP1	**<0.001**	**0.011**	hsa-miR-141-3p / hsa-miR-16-5p / hsa-miR-548ab / hsa-miR-548ak
GNB2	**<0.001**	**0.014**	hsa-miR-331-3p / hsa-miR-16-5p / hsa-miR-766-5p
MTHFD2	**<0.001**	**0.014**	hsa-miR-16-5p / hsa-miR-548ab / hsa-miR-548ak / hsa-miR-625-5p / hsa-miR-20b-3p
TCFL5	**<0.001**	**0.014**	hsa-miR-331-3p / hsa-miR-16-5p
CDKN2D	**<0.001**	**0.017**	hsa-miR-331-3p / hsa-miR-451a
CLDN10	**0.001**	**0.017**	hsa-miR-331-3p / hsa-miR-486-5p
DHX8	**0.001**	**0.017**	hsa-miR-16-5p / hsa-miR-331-3p
EARS2	**0.001**	**0.017**	hsa-miR-16-5p / hsa-miR-331-3p
HIST1H2BC	**0.001**	**0.017**	hsa-miR-625-5p / hsa-miR-16-5p
PANX1	**0.001**	**0.017**	hsa-miR-16-5p / hsa-miR-548ak / hsa-miR-548ab
PHB2	**0.000**	**0.017**	hsa-miR-766-5p / hsa-miR-548ak / hsa-miR-548ab / hsa-miR-141-3p / hsa-miR-625-5p
PPIF	**0.001**	**0.017**	hsa-miR-16-5p / hsa-miR-766-5p / hsa-miR-625-5p
RARS	**0.001**	**0.017**	hsa-miR-16-5p / hsa-miR-331-3p
RPS12	**0.001**	**0.017**	hsa-miR-16-5p / hsa-miR-331-3p
TIA1	**0.001**	**0.017**	hsa-miR-16-5p / hsa-miR-331-3p
ZNRF2	**0.001**	**0.017**	hsa-miR-16-5p / hsa-miR-486-5p / hsa-miR-548ak / hsa-miR-548ab
MKI67	**0.001**	**0.017**	hsa-miR-16-5p / hsa-miR-331-3p / hsa-miR-548ak / hsa-miR-548ab
ZMPSTE24	**0.001**	**0.018**	hsa-miR-141-3p / hsa-miR-548ab / hsa-miR-548ak

Abbreviations: *PSMD11*, proteasome 26S subunit, non-ATPase 11; *DMAP1*, DNA methyltransferase 1 associated protein 1; *FHIT*, fragile histidine triad diadenosine triphosphatase; *LAMTOR4*, late endosomal/lysosomal adaptor, MAPK and MTOR activator 4; *SMPD4*, sphingomyelin phosphodiesterase 4; *TMED1*, transmembrane P24 trafficking protein 1; *USP53*, ubiquitin specific peptidase 53; *YAP1*, Yes1 associated transcriptional regulator; *GNB2*, G protein subunit beta 2; *MTHFD2*, methylenetetrahydrofolate dehydrogenase (NADP+ dependent) 2; *TCFL5*, transcription factor like 5; *CDKN2D*, cyclin dependent kinase inhibitor 2D; *CLDN10*, claudin 10; *DHX8*, DEAH-box helicase 8; *EARS2*, glutamyl-tRNA synthetase 2; *HIST1H2BC*, H2B clustered histone 4; *PANX1*, pannexin 1; *PHB2*, prohibitin 2; *PPIF*, peptidylprolyl isomerase F; *RARS*, arginyl-tRNA synthetase 1; *RPS12*, ribosomal protein S12; *TIA1*, TIA1 cytotoxic granule associated RNA binding protein; *ZNRF2*, zinc and ring finger 2; *MKI67*, marker of proliferation Ki-67; *ZMPSTE24*, zinc metallopeptidase STE24

Supplementary Data S7. Pathway enrichment and annotation analysis of differentially expressed miRNAs between people with HIV (PWH) with or without cirrhosis (LSM≥12.5kPa) one year after completion of HCV therapy.

**Pathways**	**Enrichment**	**P-value**	**miRNAs**
Glyoxylate and dicarboxylate metabolism	enriched	**0.002**	hsa-miR-486-5p; hsa-miR-20b-3p; hsa-miR-766-5p; hsa-miR-16-5p; hsa-miR-625-5p
Protein digestion and absorption	enriched	**0.007**	hsa-miR-20b-3p; hsa-miR-766-5p; hsa-miR-16-5p; hsa-miR-625-5p; hsa-miR-605-3p; hsa-miR-331-3p
Valine, leucine and isoleucine degradation	enriched	**0.009**	hsa-miR-486-5p; hsa-miR-20b-3p; hsa-miR-16-5p
beta-Alanine metabolism	enriched	**0.009**	hsa-miR-20b-3p; hsa-miR-766-5p; hsa-miR-16-5p
Fc gamma R-mediated phagocytosis	enriched	**0.010**	hsa-miR-486-5p; hsa-miR-20b-3p; hsa-miR-766-5p; hsa-miR-16-5p; hsa-miR-548ab; hsa-miR-625-5p; hsa-miR-605-3p; hsa-miR-331-3p; hsa-miR-451a; hsa-miR-548ak
Phosphatidylinositol signaling system	enriched	**0.010**	hsa-miR-486-5p; hsa-miR-20b-3p; hsa-miR-766-5p; hsa-miR-16-5p; hsa-miR-548ab; hsa-miR-625-5p; hsa-miR-605-3p; hsa-miR-331-3p; hsa-miR-548ak; hsa-miR-141-3p
Spliceosome	enriched	**0.010**	hsa-miR-486-5p; hsa-miR-20b-3p; hsa-miR-766-5p; hsa-miR-16-5p; hsa-miR-548ab; hsa-miR-625-5p; hsa-miR-605-3p; hsa-miR-331-3p; hsa-let-7b-3p; hsa-miR-548ak
Olfactory transduction	enriched	**0.012**	hsa-miR-20b-3p; hsa-miR-766-5p; hsa-miR-16-5p; hsa-miR-548ab; hsa-miR-625-5p; hsa-miR-605-3p; hsa-miR-331-3p; hsa-miR-548ak
One carbon pool by folate	enriched	**0.014**	hsa-miR-20b-3p; hsa-miR-766-5p; hsa-miR-16-5p; hsa-miR-548ab; hsa-miR-625-5p; hsa-miR-548ak
Other glycan degradation	enriched	**0.029**	hsa-miR-20b-3p; hsa-miR-766-5p
Glutathione metabolism	enriched	**0.030**	hsa-miR-766-5p; hsa-miR-16-5p; hsa-miR-625-5p; hsa-miR-605-3p; hsa-miR-331-3p
Allograft rejection	enriched	**0.032**	hsa-miR-486-5p; hsa-miR-16-5p; hsa-miR-625-5p; hsa-miR-605-3p
Huntington disease	enriched	**0.032**	hsa-miR-486-5p; hsa-miR-20b-3p; hsa-miR-766-5p; hsa-miR-16-5p; hsa-miR-548ab; hsa-miR-625-5p; hsa-miR-605-3p; hsa-miR-331-3p; hsa-let-7b-3p; hsa-miR-548ak; hsa-miR-141-3p
Platelet activation	enriched	**0.032**	hsa-miR-486-5p; hsa-miR-20b-3p; hsa-miR-766-5p; hsa-miR-16-5p; hsa-miR-548ab; hsa-miR-625-5p; hsa-miR-605-3p; hsa-miR-331-3p; hsa-miR-451a; hsa-miR-548ak; hsa-miR-141-3p
Tight junction	enriched	**0.032**	hsa-miR-486-5p; hsa-miR-20b-3p; hsa-miR-766-5p; hsa-miR-16-5p; hsa-miR-548ab; hsa-miR-625-5p; hsa-miR-605-3p; hsa-miR-331-3p; hsa-miR-451a; hsa-miR-548ak; hsa-miR-141-3p
Bacterial invasion of epithelial cells	enriched	**0.044**	hsa-miR-486-5p; hsa-miR-766-5p; hsa-miR-16-5p; hsa-miR-625-5p; hsa-miR-605-3p; hsa-miR-331-3p; hsa-miR-141-3p

Cardiac muscle contraction	enriched	**0.044**	hsa-miR-20b-3p; hsa-miR-766-5p; hsa-miR-16-5p; hsa-miR-625-5p; hsa-miR-605-3p; hsa-miR-331-3p; hsa-miR-141-3p
ECM-receptor interaction	enriched	**0.044**	hsa-miR-20b-3p; hsa-miR-766-5p; hsa-miR-16-5p; hsa-miR-548ab; hsa-miR-625-5p; hsa-miR-605-3p; hsa-let-7b-3p; hsa-miR-548ak
Lysine degradation	enriched	**0.044**	hsa-miR-20b-3p; hsa-miR-766-5p; hsa-miR-16-5p; hsa-miR-548ab; hsa-miR-625-5p; hsa-miR-331-3p; hsa-miR-548ak
Malaria	enriched	**0.044**	hsa-miR-486-5p; hsa-miR-20b-3p; hsa-miR-766-5p; hsa-miR-16-5p; hsa-miR-625-5p; hsa-miR-605-3p; hsa-miR-451a; hsa-miR-141-3p
Phagosome	enriched	**0.044**	hsa-miR-20b-3p; hsa-miR-766-5p; hsa-miR-16-5p; hsa-miR-625-5p; hsa-miR-605-3p; hsa-miR-331-3p; hsa-miR-451a
Pyruvate metabolism	enriched	**0.044**	hsa-miR-20b-3p; hsa-miR-766-5p; hsa-miR-16-5p; hsa-miR-625-5p; hsa-miR-605-3p; hsa-miR-331-3p; hsa-let-7b-3p
Viral myocarditis	enriched	**0.044**	hsa-miR-486-5p; hsa-miR-766-5p; hsa-miR-16-5p; hsa-miR-625-5p; hsa-miR-605-3p; hsa-miR-331-3p; hsa-let-7b-3p
Asthma	enriched	**0.044**	hsa-miR-486-5p; hsa-miR-766-5p; hsa-miR-625-5p
Base excision repair	enriched	**0.044**	hsa-miR-486-5p; hsa-miR-16-5p; hsa-miR-625-5p
Glycosaminoglycan biosynthesis - keratan sulfate	enriched	**0.044**	hsa-miR-766-5p; hsa-miR-16-5p; hsa-miR-625-5p
Mannose type O-glycan biosynthesis	enriched	**0.044**	hsa-miR-766-5p; hsa-miR-16-5p; hsa-miR-625-5p
Phototransduction	enriched	**0.044**	hsa-miR-766-5p; hsa-miR-16-5p; hsa-miR-625-5p
Primary immunodeficiency	enriched	**0.044**	hsa-miR-486-5p; hsa-miR-16-5p; hsa-miR-625-5p
Inositol phosphate metabolism	enriched	**0.047**	hsa-miR-486-5p; hsa-miR-20b-3p; hsa-miR-766-5p; hsa-miR-16-5p; hsa-miR-548ab; hsa-miR-605-3p; hsa-miR-331-3p; hsa-miR-548ak; hsa-miR-141-3p
Nucleocytoplasmic transport	enriched	**0.047**	hsa-miR-20b-3p; hsa-miR-766-5p; hsa-miR-16-5p; hsa-miR-548ab; hsa-miR-625-5p; hsa-miR-605-3p; hsa-miR-331-3p; hsa-miR-548ak; hsa-miR-141-3p
Proteasome	enriched	**0.047**	hsa-miR-20b-3p; hsa-miR-766-5p; hsa-miR-16-5p; hsa-miR-548ab; hsa-miR-625-5p; hsa-miR-605-3p; hsa-miR-331-3p; hsa-miR-548ak; hsa-miR-141-3p
Systemic lupus erythematosus	enriched	**0.047**	hsa-miR-486-5p; hsa-miR-766-5p; hsa-miR-16-5p; hsa-miR-548ab; hsa-miR-625-5p; hsa-miR-605-3p; hsa-miR-331-3p; hsa-miR-548ak; hsa-miR-141-3p
Arachidonic acid metabolism	enriched	**0.049**	hsa-miR-486-5p; hsa-miR-766-5p; hsa-miR-16-5p; hsa-miR-331-3p
Other types of O-glycan biosynthesis	enriched	**0.049**	hsa-miR-20b-3p; hsa-miR-766-5p; hsa-miR-16-5p; hsa-miR-141-3p

Supplementary Data S8. Correlations between expression of SDE miRNAs with plasma metabolite levels in PWH one year after completion of HCV therapy**. Statistics**: rho values were calculated with Spearman's correlation test. *P*-values were adjusted by FDR correction for multiple comparisons (Benjamini and Hochberg). *q*-values are shown only for correlations with |rho|>0.5.

**Metabolites**		**hsa-let-7b-3p**	**hsa-miR-10401-3p**	**hsa-miR-141-3p**	**hsa-miR-16-5p**	**hsa-miR-181a-3p**	**hsa-miR-20b-3p**	**hsa-miR-331-3p**	**hsa-miR-3940-3p**	**hsa-miR-451a**	**hsa-miR-486-5p**	**hsa-miR-548ab**	**hsa-miR-548ak**	**hsa-miR-605-3p**	**hsa-miR-625-5p**	**hsa-miR-766-5p**
**L-Methionine**	rho	0.114	0.513	0.330	0.355	-0.460	-0.379	-0.481	0.289	0.128	0.399	0.242	0.157	-0.394	-0.355	-0.457
	p-value	0.446	**<0.001**	**0.024**	**0.014**	**0.001**	**0.009**	**0.001**	**0.049**	0.390	**0.005**	0.101	0.293	**0.006**	**0.014**	**0.001**
	q-value	0.830	**0.005**	0.157	0.088	0.091	0.098	**0.035**	0.318	0.650	0.062	0.824	0.856	0.122	0.096	**0.016**
**L-Tyrosine**	rho	0.182	0.439	0.492	0.489	-0.429	-0.287	-0.469	0.248	0.276	0.514	0.333	0.331	-0.372	-0.420	-0.507
	p-value	0.220	**0.002**	**<0.001**	**<0.001**	**0.003**	0.051	**0.001**	0.093	0.060	**<0.001**	**0.022**	**0.023**	**0.010**	**0.003**	**<0.001**
	q-value	0.667	**0.027**	**0.035**	**0.008**	0.099	0.283	**0.035**	0.390	0.220	**0.004**	0.824	0.427	0.134	**0.032**	**0.005**
**L-Tryptophan**	rho	0.356	0.368	0.373	0.466	-0.179	-0.222	-0.255	0.287	0.517	0.401	0.002	0.284	-0.265	-0.504	-0.328
	p-value	**0.014**	**0.011**	**0.010**	**0.001**	0.227	0.134	0.083	0.051	**<0.001**	**0.005**	0.992	0.053	0.072	**<0.001**	**0.024**
	q-value	0.574	0.098	0.128	**0.013**	0.569	0.458	0.329	0.318	**0.008**	0.062	0.992	0.558	0.488	**0.015**	0.149
**Hypotaurine**	rho	-0.253	-0.177	-0.295	-0.578	0.093	0.221	0.321	-0.416	-0.529	-0.550	-0.134	-0.102	0.288	0.466	0.530
	p-value	0.087	0.234	**0.044**	**<0.001**	0.533	0.135	**0.028**	**0.004**	**<0.001**	**<0.001**	0.369	0.493	**0.050**	**0.001**	**<0.001**
	q-value	0.574	0.586	0.197	**0.002**	0.853	0.458	0.185	0.127	**0.008**	**0.004**	0.853	0.856	0.399	**0.015**	**0.003**
**N-(1-Deoxy-1-fructosyl) isoleucine**	rho	0.256	0.509	0.333	0.508	-0.098	-0.393	-0.332	0.278	0.419	0.486	0.017	0.323	-0.207	-0.416	-0.492
	p-value	0.083	**<0.001**	**0.022**	**<0.001**	0.510	**0.006**	**0.023**	0.059	**0.003**	**0.001**	0.910	**0.027**	0.162	**0.004**	**<0.001**
	q-value	0.574	**0.005**	0.157	**0.005**	0.850	0.098	0.165	0.318	0.053	**0.009**	0.971	0.427	0.605	**0.032**	**0.007**
**N-(1-Deoxy-1-fructosyl) methionine**	rho	0.257	0.548	0.406	0.531	-0.186	-0.372	-0.446	0.281	0.399	0.527	0.145	0.344	-0.342	-0.466	-0.565
	p-value	0.081	**<0.001**	**0.005**	**<0.001**	0.211	**0.010**	**0.002**	0.056	**0.005**	**<0.001**	0.332	**0.018**	**0.019**	**0.001**	**<0.001**
	q-value	0.574	**0.003**	0.124	**0.003**	0.561	0.100	0.044	0.318	0.073	**0.004**	0.853	0.427	0.186	**0.015**	**0.003**
	rho	0.290	0.548	0.384	0.536	-0.107	-0.338	-0.390	0.258	0.432	0.525	0.150	0.359	-0.306	-0.470	-0.540

**N-(1-Deoxy-1-fructosyl) phenylalanine**	p-value	**0.048**	**<0.001**	**0.008**	**<0.001**	0.473	**0.020**	**0.007**	0.080	**0.002**	**<0.001**	0.314	**0.013**	**0.036**	**0.001**	**<0.001**
	q-value	0.574	**0.003**	0.124	**0.003**	0.843	0.146	0.089	0.368	0.053	**0.004**	0.853	0.427	0.324	**0.015**	**0.003**
**Aspartyllysine**	rho	0.101	0.250	0.334	0.397	-0.183	-0.506	-0.316	0.233	0.097	0.342	0.144	0.248	-0.207	-0.448	-0.383
	p-value	0.501	0.090	**0.022**	**0.006**	0.217	**<0.001**	**0.030**	0.116	0.517	**0.019**	0.333	0.093	0.162	**0.002**	**0.008**
	q-value	0.852	0.410	0.157	0.054	0.561	**0.023**	0.186	0.462	0.765	0.115	0.853	0.706	0.605	**0.021**	0.070
